# P-1952. Osteoarticular Coccidioidomycosis in California: A Single-Center Experience

**DOI:** 10.1093/ofid/ofaf695.2120

**Published:** 2026-01-11

**Authors:** Radhika Arya, Daisuke Furukawa, Traci Shiu, Melissa Dzinoreva

**Affiliations:** Stanford University School of Medicine, Stanford, California; Stanford University, Palo Alto, California; Loyola University Chicago Stritch School of Medicine, Sunnyvale, California; Stanford, stanford, CA

## Abstract

**Background:**

Nearly 20,000 cases of coccidioidomycosis are reported annually in the United States. Osteoarticular involvement in coccidioidomycosis is an uncommon manifestation leading to significant morbidity, but evidence surrounding it is limited. We aimed to describe demographic and clinical patterns of osteoarticular coccidioidomycosis and identify factors associated with treatment failure to improve disease recognition and management.

**Methods:**

We performed a retrospective chart review of adults ≥18 years hospitalized with confirmed osteoarticular coccidioidomycosis at an academic tertiary care center between 2004-2021. We extracted demographic, clinical, microbiologic, treatment, and outcomes data. Univariable regression analysis was used to identify risk factors of disease progression or relapse.

**Results:**

37 patients were reviewed, of which 33 (89%) were male with median age of 49 (IQR 38-65) years and median time of follow up of 54 months (IQR 22.3-110.4). The most common sites of skeletal involvement included the spine (n=16, 43%) and knee (n=10, 27%), with pulmonary involvement (n=30, 82%) noted as the most common extra-skeletal site. 27 patients (73%) required surgical interventions, of which local amphotericin was used in 13 (48%). Monotherapy with itraconazole was the most common initial treatment regimen (n=14, 38%) followed by fluconazole (n=11, 30%), and combination therapy was used in 6 (16%). Median treatment duration was 32.3 months (IQR 13.3-66.7) with 31 (84%) patients remaining on antifungal through the last day of follow up. 11 (30%) patients experienced progression while on antifungal therapy and 9 (24%) patients experienced relapse after discontinuing antifungal. Knee infection (OR 6.8, 95% CI 1.2-38.6, p=0.03) was associated with disease progression or relapse while multiple bone involvement (OR 5.5, 95% CI 0.94-31.0, p=0.06) and prior history of coccidioidomycosis (OR 3.4, 95% CI 0.89-13.3, p=0.07) trended towards significance.

**Conclusion:**

Osteoarticular coccidioidomycosis required prolonged therapy with substantial risk of progression or relapse. Involvement of knee joint may predict poorer outcomes. Larger studies are needed to validate these associations and optimize treatment strategies.

**Disclosures:**

All Authors: No reported disclosures

